# Supplementary material for: The effects of beta-cell mass and function, intercellular coupling, and islet synchrony on Ca2+ dynamics
Source: Sci Rep. 2021 May 13;11:10268. doi: 10.1038/s41598-021-89333-x (PMC8119479; doi:10.1038/s41598-021-89333-x)
Supplement: Supplementary file 1 — Supplementary Information. [file 41598_2021_89333_MOESM1_ESM.pdf]

# Supplementary Material

## The effects of beta-cell mass and function, intercellular coupling, and islet synchrony on $Ca^{2+}$ dynamics

Maryam Saadati[1], Yousef Jamali[1*]

[1] Biomathematics Laboratory, Department of Applied Mathematics, School of Mathematical Sciences, Tarbiat Modares University,Tehran, Iran

[*] Corresponding author. Email: y.jamali@modares.ac.ir

April 17, 2021

For completeness, a detailed description of mathematical modeling is given here. The electrophysiological model of single human $\beta$-cell has been developed by Pedersen [1], and then Riz et al. included $Ca^{2+}$ dynamics in the model [2]. As mentioned in the main text, this model consists of an electrical component and a glycolytic component [3], which we describe differential equations of each component in the following.

## The electrical model

The main Equations for electrical activity, including membrane potential ($V$), submembrane $Ca^{2+}$ concentration ($Ca_{\mathrm{m}}$), and cytosolic $Ca^{2+}$ concentration ($Ca_{\mathrm{c}}$), are as follows:

$$\begin{aligned}
\frac{dV}{dt} &= -(I_{\mathrm{SK}} + I_{\mathrm{BK}} + I_{\mathrm{Kv}} + I_{\mathrm{HERGA}} + I_{\mathrm{Na}} \\
&\quad + I_{\mathrm{CaL}} + I_{\mathrm{CaPQ}} + I_{\mathrm{CaT}} + I_{\mathrm{K(ATP)}} + I_{\mathrm{leak}}), \\
\frac{dCa_{\mathrm{m}}}{dt} &= f\alpha C_{\mathrm{m}}(-I_{\mathrm{CaL}} - I_{\mathrm{CaPQ}} - I_{\mathrm{CaT}})/Vol_{\mathrm{m}} \\
&\quad - f(Vol_{\mathrm{c}}/Vol_{\mathrm{m}})\big[B(Ca_{\mathrm{m}} - Ca_{\mathrm{c}}) + (J_{\mathrm{PMCA}} - J_{\mathrm{NCX}})\big], \\
\frac{dCa_{\mathrm{c}}}{dt} &= f\big[B(Ca_{\mathrm{m}} - Ca_{\mathrm{c}}) - J_{\mathrm{SERCA}} + J_{\mathrm{leak}}\big],
\end{aligned}$$

where the intracellular free calcium concentration is estimated with these two compartments, $Ca_{\mathrm{m}}$ and $Ca_{\mathrm{c}}$.

Expressions for the ionic currents are

$$
\begin{aligned}
I_{\mathrm{SK}} &= g_{\mathrm{SK}} \frac{{Ca_{\mathrm{m}}}^n}{{K_{\mathrm{SK}}}^n + {Ca_{\mathrm{m}}}^n} (V - V_{\mathrm{K}}), \\
I_{\mathrm{BK}} &= \bar{g}_{\mathrm{BK}}\, m_{\mathrm{BK}} \left[-I_{\mathrm{Ca}}(V) + B_{\mathrm{BK}}\right] (V - V_{\mathrm{K}}), \\
I_{\mathrm{Kv}} &= g_{\mathrm{Kv}}\, m_{\mathrm{Kv}} (V - V_{\mathrm{K}}), \\
I_{\mathrm{HERG}} &= g_{\mathrm{HERG}}\, m_{\mathrm{HERG}}\, h_{\mathrm{HERG}} (V - V_{\mathrm{K}}), \\
I_{\mathrm{Na}} &= g_{\mathrm{Na}}\, m_{\mathrm{Na},\infty}(V)\, h_{\mathrm{Na}} (V - V_{\mathrm{Na}}), \\
I_{\mathrm{CaL}} &= g_{\mathrm{CaL}}\, m_{\mathrm{CaL},\infty}(V)\, h_{\mathrm{CaL}} (V - V_{\mathrm{Ca}}), \\
I_{\mathrm{CaPQ}} &= g_{\mathrm{CaPQ}}\, m_{\mathrm{CaPQ},\infty}(V) (V - V_{\mathrm{Ca}}), \\
I_{\mathrm{CaT}} &= g_{\mathrm{CaT}}\, m_{\mathrm{CaT},\infty}(V)\, h_{\mathrm{CaT}} (V - V_{\mathrm{Ca}}), \\
I_{\mathrm{K(ATP)}} &= g_{\mathrm{K(ATP)}} (V - V_{\mathrm{K}}), \\
I_{\mathrm{leak}} &= g_{\mathrm{leak}} (V - V_{\mathrm{leak}}),
\end{aligned}
$$

where

$$I_{\mathrm{Ca}}(V) = I_{\mathrm{CaL}} + I_{\mathrm{CaPQ}} + I_{\mathrm{CaT}}.$$

The gating dynamics of the ion channels are assumed to follow a first-order differential equation (X denotes the type of channels)

$$\frac{dm_{\mathrm{X}}}{dt} = \frac{m_{\mathrm{X},\infty}(V) - m_{\mathrm{X}}}{\tau_{m_{\mathrm{X}}}},$$

where $m_{\mathrm{X}}$ ($h_{\mathrm{X}}$) describes activation (inactivation) of the X channels, $\tau_{m_{\mathrm{X}}}$ ($\tau_{h_{\mathrm{X}}}$) is the time-constant of activation (inactivation), and $m_{\mathrm{X},\infty}(V)$ $\big(h_{\mathrm{X},\infty}(V)\big)$ represents the steady-state activation (inactivation) of the X channels, which depends on the membrane potential.

The steady state activation (inactivation) functions are described by sigmoidal Boltzmann functions, increasing with membrane potential:

$$m_{\mathrm{X},\infty}(V) = \frac{1}{1 + \exp[(V - V_{m_{\mathrm{X}}})/n_{m_{\mathrm{X}}}]},$$

except for inactivation of L-type calcium channels, which is supposed to be $Ca^{2+}$-dependent:

$$h_{\mathrm{CaL},\infty}(V) = \max(0, \min\{1, 1 + [m_{\mathrm{CaL},\infty}(V)(V - V_{\mathrm{Ca}})]/57\mathrm{mV}\}).$$

The voltage-dependent time constant for activation of the delayed rectifying potassium (Kv) channels is modeled as

$$\tau_{m_{\mathrm{Kv}}} = \begin{cases} \tau_{m_{\mathrm{Kv},0}} + 10 \exp\left(\dfrac{-20\mathrm{mV} - V}{6\mathrm{mV}}\right) \mathrm{ms}, & \text{for } V \geq 26.6 \text{ mV}, \\ \tau_{m_{\mathrm{Kv},0}} + 30 \text{ ms}, & \text{for } V < 26.6 \text{ mV}. \end{cases}$$

Finally, the fluxes of $Ca^{2+}$ through SERCA and PMCA pumps, and $Na^{+}/Ca^{2+}$ exchangers are given by:

$$J_{\mathrm{SERCA}} = J_{\mathrm{SERCA,max}} \frac{Ca_{\mathrm{c}}{}^2}{K_{\mathrm{SERCA}}{}^2 + Ca_{\mathrm{c}}{}^2}$$
$$J_{\mathrm{PMCA}} = J_{\mathrm{PMCA,max}} \frac{Ca_{\mathrm{m}}}{K_{\mathrm{PMCA}} + Ca_{\mathrm{m}}}$$
$$J_{\mathrm{NCX}} = J_{\mathrm{NCX,0}}\, Ca_{\mathrm{m}}.$$

A list of all the parameters for the electrical model and their values are reported in Table 1.

**Table 1:** Electrical model parameters, as reported in Ref. [4]. Default values used unless mentioned otherwise.

| Parameter | | | Parameter | | |
|---|---|---|---|---|---|
| $V_{\mathrm{K}}$ | -75 | mV | $V_{\mathrm{Na}}$ | 70 | mV |
| $V_{\mathrm{Ca}}$ | 65 | mV | $V_{\mathrm{leak}}$ | -30 | mV |
| $g_{\mathrm{SK}}$ | 0.030 | nS/pF | $K_{\mathrm{SK}}$ | 0.57 | µM |
| n | 5.2 | | | | |
| $\bar{g}_{\mathrm{BK}}$ | 0.020 | nS/pA | $\tau_{m_{\mathrm{BK}}}$ | 2 | ms |
| $V_{m_{\mathrm{BK}}}$ | 0 | mV | $n_{m_{\mathrm{BK}}}$ | -10 | mV |
| $B_{\mathrm{BK}}$ | 20 | pA/pF | | | |
| $g_{\mathrm{Kv}}$ | 0.215 | nS/pF | $\tau_{m_{\mathrm{Kv,0}}}$ | 2 | ms |
| $V_{m_{\mathrm{Kv}}}$ | 0 | mV | $n_{m_{\mathrm{Kv}}}$ | -10 | mV |
| $g_{\mathrm{HERG}}$ | 0 | nS/pF | | | |
| $V_{m_{\mathrm{HERG}}}$ | -30 | mV | $n_{m_{\mathrm{HERG}}}$ | -10 | mV |
| $V_{h_{\mathrm{HERG}}}$ | -42 | mV | $n_{h_{\mathrm{HERG}}}$ | 17.5 | mV |
| $\tau_{m_{\mathrm{HERG}}}$ | 100 | ms | $\tau_{h_{\mathrm{HERG}}}$ | 50 | ms |
| $g_{\mathrm{Na}}$ | 0.400 | nS/pF | $\tau_{h_{\mathrm{Na}}}$ | 2 | ms |
| $V_{m_{\mathrm{Na}}}$ | -18 | mV | $n_{m_{\mathrm{Na}}}$ | -5 | mV |
| $V_{h_{\mathrm{Na}}}$ | -42 | mV | $n_{h_{\mathrm{Na}}}$ | 6 | mV |
| $g_{\mathrm{CaL}}$ | 0.140 | nS/pF | $\tau_{h_{\mathrm{CaL}}}$ | 20 | ms |
| $V_{m_{\mathrm{CaL}}}$ | -25 | mV | $n_{m_{\mathrm{CaL}}}$ | -6 | mV |
| $g_{\mathrm{CaPQ}}$ | 0.170 | nS/pF | | | |
| $V_{m_{\mathrm{CaPQ}}}$ | -10 | mV | $n_{m_{\mathrm{CaPQ}}}$ | -10 | mV |
| $g_{\mathrm{CaT}}$ | 0.050 | nS/pF | $\tau_{h_{\mathrm{CaT}}}$ | 7 | ms |
| $V_{m_{\mathrm{CaT}}}$ | -40 | mV | $n_{m_{\mathrm{CaT}}}$ | -4 | mV |
| $V_{h_{\mathrm{CaT}}}$ | -64 | mV | $n_{h_{\mathrm{CaT}}}$ | 8 | mV |
| $g_{\mathrm{leak}}$ | 0.015 | nS/pF | | | |
| $J_{\mathrm{SERCA,max}}$ | 0.150 | µM/ms | $K_{\mathrm{SERCA}}$ | 0.27 | µM |
| $J_{\mathrm{PMCA,max}}$ | 0.021 | µM/ms | $K_{\mathrm{PMCA}}$ | 0.50 | µM |
| $J_{\mathrm{NCX,0}}$ | 0.01867 | $\mathrm{ms}^{-1}$ | $J_{\mathrm{leak}}$ | 0.00094 | µM/ms |
| $f$ | 0.01 | | $\alpha$ | $5.18 \times 10^{-15}$ | µmol/pA/ms |
| $C_{\mathrm{m}}$ | 10 | pF | $Vol_{\mathrm{m}}$ | 0.1 | pL |
| $B$ | 0.1 | $\mathrm{ms}^{-1}$ | $Vol_{\mathrm{c}}$ | 1.15 | pL |
| $g_{\mathrm{c}}$ | 0.01 | nS/pF | | | |

# The glycolytic model

The model is based on data from the key enzymes of the glycolytic pathway in the human $\beta$-cells, playing an important role in the oscillatory behavior of the glycolysis. The glycolytic dynamics in this model primarily depend on the activation of the enzyme phosphofructokinase (PFK) by one of its products, fructose-1,6-bisphosphate (FBP). The glycolytic equations are

$$\frac{dG6P.F6P}{dt} = V_{\mathrm{GK}} - V_{\mathrm{PFK}},$$
$$\frac{dFBP}{dt} = V_{\mathrm{PFK}} - V_{\mathrm{FBA}},$$
$$\frac{dDHAP.G3P}{dt} = 2V_{\mathrm{FBA}} - V_{\mathrm{GAPDH}},$$

where $V_{\mathrm{GK}}$ is the reaction rate of glucokinase enzyme, resulting in the formation of glucose-6-phosphate (G6P) which is converted to fructose 6-phosphate (F6P) by glucose-6-phosphate isomerase, a process assumed to be in rapid equilibrium. The sum of G6P and F6P is denoted $G6P.F6P$. $V_{\mathrm{PFK}}$ is the reaction rate of PFK producing FBP, which is removed through the fructose-bisphosphate aldolase (FBA) catalyzed reaction, which produces dihydroxyacetone-phosphate (DHAP) and glyceraldehyde-3-phosphate (G3P) with the rate $V_{\mathrm{FBA}}$. DHAP is assumed to be in equilibrium with G3P, and $DHAP.G3P$ is the sum of DHAP and G3P. Finally, G3P is removed by glyceraldehyde-3-phosphate dehydrogenase (GAPDH) with the rate $V_{\mathrm{GAPDH}}$.

Expressions for the reaction rates are:

$$V_{\mathrm{GK}} = V_{\mathrm{GK,max}} \frac{G^{\mathrm{hGK}}}{K_{\mathrm{GK}}^{\mathrm{hGK}} + G^{\mathrm{hGK}}},$$

$$V_{\mathrm{PFK}} = V_{\mathrm{PFK,max}} \frac{\left(\dfrac{F6P}{K_{\mathrm{PFK}}}\right)^{\mathrm{h(FBP)}}}{\left(\dfrac{F6P}{K_{\mathrm{PFK}}}\right)^{\mathrm{h(FBP)}} + \dfrac{1+\left(\dfrac{FBP}{X_{\mathrm{PFK}}}\right)^{\mathrm{hX}}}{1+\left(\dfrac{FBP}{X_{\mathrm{PFK}}}\right)^{\mathrm{hX}} \alpha_{\mathrm{G}}^{\mathrm{h(FBP)}}}},$$

$$V_{\mathrm{FBA}} = \frac{V_{\mathrm{FBA,max}}\left(\dfrac{FBP}{K_{\mathrm{FBA}}} - \dfrac{G3P \times DHAP}{P_{\mathrm{FBA}}\, Q_{\mathrm{FBA}}\, K_{\mathrm{FBA}}}\right)}{1 + \dfrac{FBP}{K_{\mathrm{FBA}}} + \dfrac{DHAP}{Q_{\mathrm{FBA}}} + \dfrac{G3P \times DHAP}{P_{\mathrm{FBA}}\, Q_{\mathrm{FBA}}}},$$

$$V_{\mathrm{GADPH}} = V_{\mathrm{GADPH,max}} \frac{G3P}{K_{\mathrm{GADPH}} + G3P},$$

where

$$F6P = (G6P.F6P)\, K_{\mathrm{GPI}} \,/\, (1 + K_{\mathrm{GPI}}),$$

$$G3P = (DHAP.G3P)\, K_{\mathrm{TPI}} \,/\, (1 + K_{\mathrm{TPI}}),$$

$$DHAP = (DHAP.G3P) - G3P,$$

and

$$h(FBP) = h_{\mathrm{PFK}} - (h_{\mathrm{PFK}} - h_{\mathrm{act}}) \frac{FBP}{K_{\mathrm{FBA}} + FBP}.$$

The enzymatic reactions of the lower part of glycolysis are supposed to be a very large limiting rate, and the GAPDH reaction rate reflects the flux through the glycolytic pathway and controls $\beta$-cell's electrophysiological behavior via an "ATP-mimetic" variable, $a$, as described by

$$\frac{da}{dt} = V_{\mathrm{GAPDH}} - k_{\mathrm{A}} a,$$

where the conductance of the K(ATP) channels depends inversely on $a$:

$$g_{\mathrm{K(ATP)}} = \hat{g}_{\mathrm{K(ATP)}}/(1 + a).$$

All parameter values for the glycolytic model indicate in Table 2.

**Table 2:** Glycolytic model parameters, as reported in Ref. [4]. Default values used unless mentioned otherwise.

| **Parameter** | | | **Parameter** | | |
|---|---|---|---|---|---|
| $V_{\mathrm{GK,max}}$ | 0.0000556 | mM/ms | $K_{\mathrm{GK}}$ | 8.0 | mM |
| hGK | 1.7 | | $G$ | 10 | mM |
| $V_{\mathrm{PFK,max}}$ | 0.000556 | mM/ms | $K_{\mathrm{PFK}}$ | 4.0 | mM |
| $h_{\mathrm{PFK}}$ | 2.5 | | $h_{\mathrm{act}}$ | 1 | |
| $X_{PFK}$ | 0.01 | mM | hX | 2.5 | |
| $\alpha_{\mathrm{G}}$ | 5.0 | | | | |
| $V_{\mathrm{FBA,max}}$ | 0.000139 | mM/ms | $K_{\mathrm{FBA}}$ | 0.005 | mM |
| $P_{\mathrm{FBA}}$ | 0.5 | mM | $Q_{\mathrm{FBA}}$ | 0.275 | mM |
| $V_{\mathrm{GADPH,max}}$ | 0.00139 | mM/ms | $K_{\mathrm{GADPH}}$ | 0.005 | mM |
| $K_{\mathrm{GPI}}$ | 0.3 | | $K_{\mathrm{TPI}}$ | 0.045455 | |
| $k_{\mathrm{A}}$ | 0.0001 | $\mathrm{ms}^{-1}$ | $\hat{g}_{\mathrm{K(ATP)}}$ | 0.050 | nS/pF |
| $P_{\mathrm{G6P.F6P}}$ | 0.01 | $\mathrm{ms}^{-1}$ | | | |

# References


[1] Pedersen, M.G. (2010). A biophysical model of electrical activity in human $\beta$-cells. Biophys J **99(10)**, 3200-3207. doi:10.1016/j.bpj.2010.09.004.

[2] Riz, M., Braun, M., and Pedersen, M.G. (2014). Mathematical modeling of heterogeneous electrophysiological responses in human $\beta$-cells. PLoS Comput Biol **10(1)**:e1003389. doi:10.1371/journal.pcbi.1003389.

[3] Westermark, P.O., and Lansner, A. (2003). A model of phosphofructokinase and glycolytic oscillations in the pancreatic $\beta$-cell. Biophys J **85(1)**, 126-139. doi:10.1016/S0006-3495(03)74460-9.

[4] Loppini, A., Braun, M., Filippi, S., and Pedersen, M.G. (2015). Mathematical modeling of gap junction coupling and electrical activity in human $\beta$-cells. Phys Biol **12(6)**:066002. doi:10.1088/1478-3975/12/6/066002.
